# Correction to “Comparing functional and genomic‐based precision medicine in blood cancer patients”

**DOI:** 10.1002/hem3.70220

**Published:** 2025-10-08

Kazianka L, Pichler A, Agreiter C, et al. Comparing functional and genomic‐based precision medicine in blood cancer patients. *HemaSphere*. 2025;9(4):e70129. doi:10.1002/hem3.70129

A funder was not acknowledged in the original article. The following statement has now been added in the Funding section: “Open Access funding provided by Medizinische Universitat Wien/KEMÖ.”

The original article has been updated. We apologize for this error.

